# Early-stage studies to larger-scale trials: investigators’ perspectives on scaling-up childhood obesity interventions

**DOI:** 10.1186/s40814-022-00991-8

**Published:** 2022-02-07

**Authors:** L. von Klinggraeff, R. Dugger, A. D. Okely, D. Lubans, R. Jago, S. Burkart, R. G. Weaver, B. Armstrong, C. D. Pfledderer, M. W. Beets

**Affiliations:** 1 Arnold School of Public Health, University of South Carolina, 921 Assembly Street, Room 130, Columbia, SC 29205 USA; 2 School of Health and Society, University of Wollongong, Wollongong, Australia; 3 Priority Research Centre for Physical Activity and Nutrition, University of Newcastle, Callaghan, Australia; 4 Centre for Exercise, Nutrition & Health Sciences, School for Policy Studies, University of Bristol, Bristol, UK

**Keywords:** Childhood obesity, Scalability, Intervention, Translation, Pilot

## Abstract

**Background:**

Pilot/feasibility studies play an important role in the development and refinement of behavioral interventions by providing information about feasibility, acceptability, and potential efficacy. Despite their importance and wide-spread use, the approaches taken by behavioral scientists to scale-up early-stage studies to larger-scale trials has received little attention. The aim of our study was to understand the role that pilot studies play in the development and execution of larger-scale trials.

**Methods:**

We conducted interviews with childhood obesity researchers who had published pilot behavioral interventions and larger-scale trials of the same or similar interventions. Questions were asked about the role of pilot studies in developing larger-scale trials and the challenges encountered when scaling-up an intervention based upon pilot findings. Data were coded and analyzed using an inductive analytic approach to identify themes.

**Results:**

Twenty-four interventionists (54% women, 37–70 years old, mean 20 years since terminal degree) completed a total of 148 pilot studies across their careers (mean 6.4, range 1–20), of which 59% were scaled-up. Scaling was described as resource intensive and pilot work was considered essential to successfully competing for funding by 63% of the sample (*n* = 15). When asked to define a high-quality pilot study, interventionists described studies that allowed them to evaluate two independent factors: components of their intervention (e.g., acceptability, feasibility) and study parameters (e.g., sample size, measures). Interventionists expressed that more process implementation measures, different study designs, and additional iterations could improve decisions to scale-up. Most agreed that pilot studies were likely to produce inflated estimates of potential efficacy though only nine interventionists provided potential solutions for decreasing inflated measures of efficacy. Suggested major causes of inflated effects included high levels of oversight in pilot studies (e.g., researcher support), reliance on subjective measures, and utilizing convenience or highly motivated samples. Potential solutions included designing pilots for real-world implementation, only conducting randomized controlled pilot studies, and pre-registering pilot studies.

**Conclusions:**

Pilot studies purposes are multifaceted and deemed essential to obtaining funding for larger-scale trials. Clarifying the form and function of preliminary, early-stage research may enhance the productive utilization of early-stage studies and reduced drops in efficacy when transitioning to larger scale studies.

**Supplementary Information:**

The online version contains supplementary material available at 10.1186/s40814-022-00991-8.

## Background

To achieve widespread improvements in non-communicable diseases rates, interventions shown to be efficacious in smaller-scale studies need to be effective when “scaled-up” for widespread impact [1–5]. Successful implementation of behavioral interventions at-scale is a matter of international importance [6–10], though successful execution of effective scaling in behavioral health is difficult [11]. Recent reviews have revealed a frequent pattern whereby scaled-up interventions that have assessed for effectiveness at the pilot/feasibility stage often produce non-significant and/or substantially smaller effects compared to earlier pilot/feasibility trials [1, 12–14]. This may be due in part to a repeated pattern of “failure to scale”, “voltage drop”, or “scale-up penalty.” These factors have been consistently documented, indicating a need for increased attention to the early stage of intervention development and testing. Failure of behavioral interventions to effectively scale-up wastes valuable resources and significantly delays progress in reducing non-communicable diseases. This phenomenon represents a critical barrier to progress in developing effective behavioral interventions that perform once scaled-up.

Though the terminology and purpose of preliminary early-stage studies, commonly referred to as pilot or feasibility studies is debated [15–17], they are generally acknowledged as a fundamental and critical step in the process of intervention development [18, 19]. Researchers use pilot studies to prepare for a larger-scale iteration of a same or similar intervention by providing conceptual clarity (i.e., “proof of concept”), “optimization” of complex interventions [20], provide experience navigating potential obstacles likely to occur in intervention implementation. They also provide information about perception among participants’ that the treatment is agreeable (i.e., acceptability), intervention feasibility, evaluation feasibility, and preliminary efficacy [17, 21]. Among the many models proposed for scaling-up health interventions [19], such as the Obesity Related Behavioral Intervention Trials (ORBIT) model [18] or the NIH Stage Based Model for Behavioral Intervention Development [22] nearly all frame the implementation process as an cumulative, multi-phased process beginning with formative or pilot-related work.

How behavioral scientists actually approach intervention development from early-stage studies to larger-scale trials remains unexplored in the literature. Understanding the experiences of senior researchers who conduct pilot studies and subsequent larger trials could help elucidate the purpose, motivations, and difficulties of piloting behavioral interventions and add depth and contextual significance to our understanding of the scaling processes. A clear understanding of the purpose and function of pilot studies can help the field adopt and integrate improved practices in pilot study execution [23]. The purpose of our study was to conduct qualitative interviews with lead authors of a published pilot study that had a subsequent published larger-scale trial on a topic related to childhood obesity in order to better understand to understand, the role that pilot studies play in the development and execution of larger-scale trials.

## Methods

### Interview guide development

We developed a semi-structured interview guide using a collaborative process. Questions were aimed at eliciting participants’ personal experiences in the design and scaling of behavioral interventions and were based on the research team’s experiences as interventionists as well as theories of program implementation [24]. Questions were initially developed and reviewed by a team of content experts (RJ, DL, AO) who evaluated them in terms of clarity, content, and alignment with the research question.

A preliminary interview was conducted to ascertain the length, pace, and the suitability of question sequence for the interviews. For the preliminary interview, one individual was selected from the research team who had also provided input in question development (DL). Based on feedback from the preliminary interview, questions were amended for additional clarity and a semi-structured format was chosen to facilitate a conversational interviewing style, allowing the interviewer to follow-up with additional questions as needed. The finalized formal interview guide consisted of 10 predetermined open-ended questions.

### Recruitment

Thirty-eight previously identified authors of published pilot/feasibility studies and subsequent larger-scale trials of behavioral interventions targeting childhood obesity were eligible for participation. Procedures used to identify all possible qualifying pilot studies and their subsequent well powered trials have been described elsewhere [12].

All 38 authors were invited to participate in the study by email in November of 2019. The initial recruitment email introduced the study and the study’s purpose. Each email provided potential participants with citations for the specific pilot/feasibility and larger-scale studies that had led them to be included in the study. Emails included definitions for “pilot/feasibility study” and “behavioral intervention” to help orient participants. Participants were encouraged to respond to the email and select one of three listed time/dates for an interview lasting approximately 30 to 60 min in length. After the initial email, non-responding participants were contacted up to three more times to participate. After no response from the third contact, no more attempts to recruit participants were made.

All procedures were approved by the first author’s institutional review board (registration number Pro00086876) prior to enrollment of the first participant and were consistent with the ethical standards outlined by the Helsinki Declaration of 1975, as revised in 2000 [25].

### Data collection

#### Interviews

Interviews took place through video conference software (Zoom Video Communications, San Jose, CA) for participants living outside of the USA and/or via phone for U.S. residents. All interviews were conducted by one of the authors (MB). During the interview, the interviewer guided the conversation through the predetermined questions attempting to not make any leading statements. Questions were followed up with probing questions for clarification and to explore new ideas as necessary. All interviews were audio recorded and notes were taken throughout the interview to capture initial thoughts and observations. Participants were read an IRB-approved script which informed them of the interview’s purpose and the use of the data. Participants provided verbal consent to have their interview recorded and used for the study.

#### Transcription

All transcription was done verbatim using Otter.ai (AISense, Los Altos, CA) and verified by trained research assistants. Transcriptions were uploaded into NVivo 12 Plus (QSR International, Doncaster Australia) for coding and synthesis.

#### Analysis

Two trained research assistants (LV, RD) coded, analyzed, and synthesized the data using analytic induction [26, 27]. Both research assistants had completed formal coursework in qualitative methodology for public health research and had assisted in at least two prior, qualitative studies both as interviewers and coders.

Modified analytic induction was appropriate for this study, given that we expected interventionists who are interviewed to explain successful/unsuccessful translation of pilot to large-scale intervention results by citing factors that can be traced to both well-documented mediating variables in implementation science in addition to novel, less documented experiences expressed by participants.

This process was comprised of four key steps: data immersion, creating codes, arranging themes, and formulating and refine the explanatory hypothesis. The first step was immersion which allowed for a detailed examination of the data during which emergent themes began to surface. Once the data had been examined, the two independent coders read the transcripts in batches of 2–3 transcripts and compared coding line by line, discussing and resolving disagreements. Themes were identified a priori, and emergent themes identified during data emersion were incorporated. Themes were identified in the literature as prevalent features of scaled-up interventions [12]. Coders met weekly to review themes and discussed any disagreements until consensus was reached. The final step was to formulate and refine the explanatory hypothesis. This was done using an arrangement of codes to identify common dimensions across themes [28]. Once a preliminary explanatory hypothesis was reached, transcripts were revisited, and hypotheses redefined and revised as negative cases were detected. During the arrangement of themes and the formation of the explanatory hypotheses, the two coders debriefed with the principal investigator (MB) every other week. Debriefs included reviewing data organization, debating thematic arrangement and revisiting points of disagreement between the two coders.

#### Trustworthiness

Guided by Shenton’s provisions to ensure credibility, transferability, dependability, and confirmability in qualitative research [29], intentional and systematic methodological steps were embedded within the study to improve trustworthiness of the data collection, analysis, and synthesis. To support the credibility of the findings, consistent, scheduled peer-engagement occurred between authors during every phase of the project development and execution. This included question development (peer-scrutiny; MB, AO, DL, RJ), data collection (reflective commentary; MB, RW), and data analysis (peer-debriefing; RD, LV, MB). Our study was informed by a meta-epidemiologic study on the same population of behavioral researchers as well as work that has consistently documented the “failure to scale” phenomena [12]. This conceptual foundation supports the transferability of the findings and allows for allows for parallels to be drawn between the quantitative and qualitative data. This approach of using quantitative data as a foundation for qualitative exploration, also serves to bolster the confirmability of the findings, as does the detailed description of the analytic processes employed during data synthesis [29].

## Results

Of the 38 individuals eligible to participate, a total of 24 interviews were completed (62%). The remaining eligible participants did not respond to the email invitations. Interviews varied in length ranging from 32 to 68 min (mean 47.45). The interviews were conducted between November 2019 and March 2020. Participants predominantly self-identified as White (92%), were employed by universities (92%) and currently or formally held tenured positions (87%) with an average of 20 years of research experience (range 11–35 years). The average age of participants was 50.5 years (SD 8.8) and 46% were male. Most participants resided in Australia (35%), the USA (38%), and the UK (19%).

Through modified analytic induction 20 themes were generated containing 111 subthemes which were organized into four broad categories, or stages: (1) conceptualization of an intervention, (2) interpretation of results, (3) scaling, and (4) reflection (Table 1).

**Table 1:** Thematic analysis results

Theme	Definition	Exemplary quote	
**I. Conceptualization of intervention**			
Gathering information	Conduct an initial exploration of potential interventions including literature reviews and qualitative inquiry	1a	“I like to make sure that I'm informed by investors in the program. So if it’s a school-based program, I’d be speaking to the school, the children, the parents, or people who are experts in that area, to make sure that the program is what they are looking for, what they need.” ID6
		1b	“Looking at the evidence based behavioral strategies techniques that have been shown to be useful within that specific population, you know, highlighting the sort of the mechanisms of actions.” ID23
Measurement logistics	Will study measures result in a sufficient amount of quality data.	2a	“Do we want to do DEXA scans in this particular trial? Will people accept these DEXA? Let's try it. Let's see. Will they wear FitBits? Will they wear them 24 hours? Will they wear them at night so we can monitor what's going on at sleep? You're getting pilot usability and feasibility feedback.” ID19
		2b	“You use pilot studies to check your measurements, to be sure you can do them. The measures I do are more complicated to deliver because we’re not located near the participants.” ID5
Trial parameters	Will intervention and study design be well-received by the target population and whether study can be executed with sufficient fidelity to produce a valid test of intervention efficacy, feasibility, and acceptability	3a	“Can you recruit at the rate that you think? Will people deliver the intervention? Can we assign people to control? And is that acceptable? Testing all of those different parameters.” ID`
		3b	“You're tracking a whole range of methodological considerations, testing the actual program or intervention, and it gives you an opportunity to see the acceptability, the feasibility, of delivery, the quality” ID17
Mirror larger trial	Intentional consideration of the alignment between preliminary studies and future, larger trials of the same/similar intervention	4a	“The pilot study has to be conducted exactly the way the future large-scale study would be conducted. Naturally, it has to be conducted on a smaller scale because of the funds available at that time.” ID22
		4b	“In terms of mimicking what would actually happen in a definitive trial…sometimes you can’t know what will happen until you’ve made these mistakes.” ID10
**II. Interpretation of pilot study results**			
Process implementation measures	Measures of process implementation including fidelity and dose, as well as indicators of participant engagement such as recruitment, retainment, and qualitative feedback.	5a	“We knew we could recruit and retain. We knew the kids liked it, the staff were reporting, the engagement was high. That was key because the intervention required quite high engagement levels. We could get good physical activity data with high compliance. That was a big tip.” ID13
		5b	“I want to see some data that says, number one, people come and number two, people will stay in till the end.” ID9
Indicators of preliminary efficacy	Markers used to assess potential efficacy such as trends in primary outcomes, effects of theorized mediators, and tests of statistical significance.	6a	“Pilot studies can provide some evidence to move forward, but they are often underpowered to detect differences. So, I think pilot trials have a place, but I don’t think it should be the deciding factor.” ID2
		6b	“What we did have was some pre and post data, and a hunch that we liked it” ID4
		6c	“A pilot study has to provide some evidence of efficacy. The suggestion to conduct a feasibility or pilot study and not worry about the outcome… we could waste a lot of resources if we had that view.” ID14
		6d	It’s a bit of a conundrum. I do think that you can falsely say ‘oh, well we didn’t show an effect in our preliminary efficacy because the sample size was small, and the timing was off’. ID24
Inflated effects	Aspects of pilot studies that may inflate indicators of efficacy.	7a	“The participants in a pilot study are the cream of the crop, and when you’re doing the larger study those people have already participated.” [Ann Davis]
		7b	“In the pilot study we had teachers delivering intervention and in the main trial we went to the core non-degree staff delivering the intervention. So that probably inflated the effect of the pilot.” ID21
		7c	“There’s no point doing it, showing all this promise from a pilot study when many of the components have no chance of going to the next stage.” ID17
**Concept III: Scaling piloted interventions**			
Re-piloting interventions	Engaging in more than one round of preliminary testing to enhance intervention effects or address identifed obstacles	8a	“We had some effects, but we thought we could make them better. So we added more teaching practice and we revised the activities to make sure that they maximized physical activity opportunity.” ID3
		8b	“The participants just weren't engaging beyond our initial clinic visit and that suggested, to me, that what we had developed was probably not going to be sufficiently engaging.” ID15
		8c	“If the core aim of what you are aiming to achieve has to change substantially, then I think you should reconsider whether it warrants another pilot.” [Ralph Maddison]
Strategies	Strategies to ensure a larger trial was completed	9a	“We had very good partnerships. We were embedded in the local school boards and they saw it as very useful from both a health and an educational point of view and the larger scale trial was really just a larger scale longer version of the pilot study.” ID20
		9b	“We were mindful that the teachers in the intervention schools may not be as motivated as those in the pilot schools. So we had to give them some flexibility around how they would be implementing the intervention.” ID18
		9c	“As we moved from a pilot study to a larger-scale trial, it was not possible to get one person to train all the teachers, so we kind of had to move to a train the trainer model.” ID10
Funding	Choices made to conduct, and publish, preliminary work were based upon investigators perceptions of the difficulty, and value of the preliminary work as it related to obtaining additional funding.	10a	“The primary outcome for our larger study might be the secondary outcome for the pilot study. In our pilot stud, we might look at a behavioral outcome rather than a clinical outcome because we know that we know we are not going to show anything on the clinical outcomes” ID15
		10b	“The big incentive of having good pilot data is getting funding.” ID7
		10c	“I don’t do a pilot study unless I know it’s worthwhile.” ID6
		10d	“Journals don’t want to publish no results, and pilots are often null.” ID2
		10e	“If you focus on publications quality, then pilot study publications might not be the highest quality publications. They might not be getting the best or highest ranked journals” ID3
Adaptions	Identification of obstacles with study design or intervention protocol and brainstorms ways to ameliorate the identified problems	11a	“We received feedback from the first feasibility trial and they said this certain activity is really good. So we focused on the ones that worked really well and then the feedback that we got from the second study was that these didn’t work so well.” ID3
		11b	“The other key difference is that based on our pilot study intervention was having more of an effect on children with a propensity for overweight or obesity. So in the larger scale study, we focused specifically on those children.” ID7
		11c	“If people didn't adhere to what you're doing, if you couldn't recruit enough people, if satisfaction was a complete fail….I would have probably done adjustments along the way to make the pilot successful, but if I hadn’t, then okay, I’d need to pilot this again.” ID24
		11d	“I’d consider whether you have differential dropout or follow-up between the intervention and control groups. And if you do, then you will need to see how best to address that before moving on to the full trial.” ID13
Challenges	Challenges encountered when scaling pilot studies	12a	“In the pilot we had a small amount of group of teachers who were very motivated and who want to bring about a change but in our larger trial we had multiple teachers, multiple schools. So staff felt like their principal or their head of department had a sort of top-down approach.” ID18
		12b	“The control group was more powerful than we expected and the intervention didn’t look that meaningful next to the control group. That made it challenging argue for the larger study.” ID5
		12c	“For the larger study, going into low income schools in more disadvantaged areas, recruiting, retaining, and gauging is just so much harder.” ID14
**Concept IV: Reflection**			
Lessons learned	The process of executing a pilot study, and subsequently larger trial, provided investigators with valuable insight	13a	“We’ll sit there in a room for a day, go through things, and ask ‘What do you think?’ and then say ‘oh well that didn’t work, this didn’t work.’ So we put our minds together and, use those lessons from our other trials, to move forward.” ID6
		13b	“I think you’ve got to have the methodological questions built in and you might also want to do a much more detailed evaluation of behavior changes rather than just focusing on the outcome which you know in a larger trial you might not be able to collect.” ID4
		13c	“My lesson was to not sit there too long and try to do multiple feasibility studies.” ID11
		13d	“I learned a lot about just this notion of the need to not overreach and not overthink what it is that you can do.” ID19
		13e	“We know now that a big study has to be very simple.” ID12
		13f	“My colleagues were proposing that we shouldn’t even test the behavioral intervention. They wanted to test use a sham intervention to test other components, such as recruitment, and blinding, and different factors. But I argued that I wanted to match the methods of the feasibility trial” ID5
Failure to scale	Combination of factors that blocked a larger scale trial based upon pilot results	14a	“The pilot studies did not go on to a full trial because the effects were not substantial enough, and not consistent enough.” ID3
		14b	“The larger trial never materialized because we were unable to get funding. We just felt like the evidence wasn’t as convincing as we would have liked.” ID18
		14c	“I wouldn't take it forward to full trial because it just wasn't well received. We didn't get good engagement with clinicians and we struggled to recruit, despite spending a lot of time and a lot of resource.” ID15

### Category 1: conceptualization of an intervention

Across the interviews, pilot studies were discussed as playing a valuable role in molding concepts and approaches into an intervention that could be scaled-up. This involved critically examining and solidifying ideas into processes or protocols that could be executed, as well as choosing target outcomes, participant populations, and measurement tools. The choice to conduct early intervention development work was driven by a desire to establish a convincing case for further resource investments in a later, larger-scale trial. Investigators hoped to provide indications that the investigators could execute the logistics of the trial (e.g., recruit participants, deliver intervention, measure outcomes), while simultaneously providing evidence that the intervention has potential impact on either primary or secondary (i.e., intermediary) outcomes, referred to as a preliminary signal. The following elements were identified by interviewees as metrics that would provide valuable information for later interpretation when designing a pilot study, prior to scaling-up an intervention.

#### Gathering information

In the initial design of the interventions, researchers sought multiple sources of information to inform intervention components. These included reviews of the literature and input from key stakeholders to inform specific intervention content (quote 1a). Emphasis was placed on generating new or novel ideas for testing, the integration of existing evidence-based practices, and targeting the mechanisms of behavior change (quote 1b).

#### Measurement logistics

Pilot studies were described as an opportunity to determine whether study measures would result in sufficient amounts of quality data. For instance, investigators described wanting to gauge whether participants would tolerate particular measures, such as dual X-ray absorptiometry, return rates of measurement tools (e.g., accelerometers), complete self-report questionnaires, determine if measures were age appropriate, and/or refine the content/focus of certain measures (e.g., survey redesign; quote 2a). Study specific contexts drove this component of the pilot study, such as delivering mHealth interventions where measures would be completed without direct contact with research staff (quote 2b).

#### Trial parameters

A key indicator for moving forward with the scale-up of an intervention was positive results on trial-related parameters. Trial-related parameters were described as target population recruitment and whether they could be recruited in sufficient numbers, retention of participants over the duration of the intervention (i.e., low attrition rates) and participant engagement in the intervention-related activities (e.g., attend the number of prescribed sessions) to receive an adequate dose for an effect. Receptivity of being randomized to conditions was also considered (quote 3a). Other trial-related parameters involved process implementation measures such feasibility and fidelity that were embedded within pilot studies, along with measures of acceptability and satisfaction (quote 3b) from both participants, and where appropriate, delivery agents.

#### Mirror larger trial

Making an intentional effort to design pilot studies a priori as if they were smaller versions of the anticipated larger, future trial was consistently mentioned. Several investigators were adamant that trial and intervention protocols be as close as possible to the future larger-scale, more well-powered trial (*n* = 3; quote 4a) while others placed emphasis on aligning conceptual components between the pilot and the larger-scale trial. This included hypothesized mechanisms of behavior change (e.g., components of behavior change theories) or intermediary behaviors related to outcomes of interest (e.g., targeting physical activity to decrease BMIz) (*n* = 8; quote 1b) because those components were within the control of the investigator (quote 4b).

### Category 2: interpretation of pilot study results

To determine if an intervention demonstrated preliminary efficacy and warranted further testing in a larger trial, interviewees discussed using multiple sources of information. These included balancing the evidence of positive results on trial-related parameters from measures of implementation and acceptability, while also considering the changes in the outcomes collected. The following components were identified as key sources of information related to interpreting the results of the pilot studies.

#### Process implementation measures

Interviewees described fidelity markers, such as the ease of training intervention personnel and the consistency with which manuals and protocols were delivered to participants as key markers of whether the intervention could be delivered as designed or if modifications were necessary. Measures of dose (e.g., number of sessions attended, adherence to intervention materials) and participant engagement (e.g., satisfaction, enjoyment) were also considered crucial sources of information for decision making from pilot studies (quote 5b). Investigators also sought evidence of favorable participant reception including qualitative feedback (quote 5a).

#### Indicators of preliminary efficacy

Opinions varied on the importance and appropriate use of hypothesis testing and statistical significance when interpreting evidence of efficacy in pilot studies. When expressing their personal views, interviewees tended to view statistical testing as either inappropriate, or not a primary concern, relying instead on intuition in conjunction with the direction and magnitude of the effects along with process measures to make decisions about judging the value of an intervention and whether it should be scaled-up (quotes 6a, b). When commenting on perceived external expectations from grant or manuscript reviewers about inferential statistics in interpreting pilot study outcomes, authors felt expectations were confusing or impractical (quotes 6c). Setting a priori thresholds for effect sizes was mentioned by several participants (*n* = 5) though specific benchmarks were not provided. Several interviewees commented that interpreting preliminary statistical tests were prone to biased interpretation (quote 6d).

#### Inflated effects

Most interviewees agreed the effects from pilot studies could be inflated. Proposed reasons for inflation included recruiting motivated samples, using highly trained delivery agents, not having a control group, and increased researcher control over all aspects of the intervention delivery (quote 7a). Only nine interventionists provided potential solutions. Solutions to address inflated effects focused on designing pilot studies to more closely resemble the conditions under which the larger-scale trial would be conducted. For example, if the classroom teachers will be the delivery agents in the larger-scale trial, then the pilot should also have the classroom teachers deliver it (quote 7b, c).

### Category 3: scaling-up piloted interventions

Once pilot testing was complete, interventions were often implemented on a larger scale. While some elements of the pilot interventions and trial parameters were maintained in the next phase of research, many were adapted. It was not uncommon for investigators to report changing the duration, typically from a shorter pilot (e.g., 12 weeks) to longer timeframe in the larger-scale trial (e.g., 6 months) or reducing the intensity of the intervention in the larger-scale trial from what was provided in the pilot. Notably, some investigators indicated using pilot studies to evaluate the logistics of deploying and collecting measures, though a challenge repeatedly mentioned when discussing larger-scale trials was the ability to collect measures with a substantially larger number of participants.

#### Re-piloting interventions

Investigators were asked to describe, either from their own experience or hypothetically, circumstances where re-piloting an intervention would be necessary based upon the results of a pilot study. Some investigators expressed rarely or never re-piloting studies (*n* = 3), while others (*n* = 5) mentioned re-piloting interventions in hopes of achieving stronger effects on their specified outcomes (quote 8a). Complete lack of engagement, inability to recruit participants, and substantial changes to intervention content were noted as signs that re-piloting was not worthwhile (quote 8b, c).

#### Strategies for scaling-up

Investigators described strategies that helped take their pilot study to larger-scale trials. These included having strong collaborative relationships with community partners (quote 9a), allowing for more flexibility during intervention implementation (quote 9b) as well as adapting the dissemination model for an increased number of participants or delivery agents (quote 9c).

#### Funding of larger-scale trials

Funding played an essential role in the execution of both pilot studies and subsequent larger-scale studies (*n* = 18). Funding was described as necessary for conducting to a pilot study, with outcomes from pilot studies deemed critical to securing additional funds to conduct a larger-scale trial of the same or similar intervention (quote 10b). Thus, pilot studies were consistently viewed as a necessary prerequisite to obtaining funding for larger-scale trials. Some interviewees believed that publishing pilot studies increased their odds of successfully obtaining large-scale funding (*n* = 9). Conversely, an equal number believed that publishing pilot studies was a difficult and disincentivized process, because pilot studies were unlikely to produce significant effects or were considered lower quality studies (quote 10d, e). Other strategies for successfully obtaining funding included conducting only pilots perceived to have a greater likelihood of success at obtaining larger-scale funding (10c) and choosing primary outcomes in pilot studies likely to indicate positive change (change quote 10a).

#### Adaptations

Intervention components and study designs often differed between the pilot and larger-scale trials (*n* = 17). Pilot study outcomes were mentioned as useful in the identification of areas for improvement when taking the intervention to scale. Notably, investigators mentioned changing intervention intensity, duration, and study design (e.g., including randomization). These changes were driven by participant feedback in the pilot studies, funding priorities, methodological requirements of larger-scale trials, or the desire to increase efficacy of the intervention (quotes 11a–11c). Insufficient recruitment, retention, or participant satisfaction (quotes 5a, 5b) were indicative of intervention adaptations, in addition to addressing practical limitations of space (e.g., school classrooms), personnel (e.g., classroom teacher aides), intervention protocol, and material (quotes 5c, 5d) when delivering the intervention to a larger audience.

#### Challenges

Most investigators encountered challenges when scaling-up their pilot studies (*n* = 18). Common challenges encountered included decreased stakeholder engagement, changes in delivery personnel, and logistical difficulties collecting outcome measures (quotes 12a–12c) in a substantially larger sample of participants. According to investigators, this resulted in less meaningful results, logistical difficulties that impeded data collection, less fidelity in intervention delivery due to a change in intervention delivery agents, ultimately rendering the impact of the scaled intervention smaller than originally anticipated.

### Category 4: reflection

All interviewees reflected upon their experiences piloting behavioral interventions and the adaptations or re-piloting they employed. Pilot studies deemed successful were often adapted during scale-up while less successful pilot studies were re-piloted or abandoned. Several investigators reflected favorably upon applying adaptations to larger-scale studies, considering them necessary and advantageous. Others felt adaptations were errors or that re-piloting could have identified appropriate intervention components to test in a larger-scale trial, leading to more impactful interventions.

#### Lessons learned

The experience of scaling pilot interventions provided investigators lessons to improve future interventions (quote 13a). Several investigators mentioned including more process implementation measures in subsequent studies (quote 13b) while others prefer more pre-development or iterative pilot work (quote 13c). Designing interventions to be simple, with fewer moving parts and complexities, were mentioned as key takeaways (quote 13d, e). Several (*n* = 10) investigators highlighted the role of collaborating with colleagues in developing interventions, though they differed on the perceived benefits. Some investigators found additional contributions detracted from intervention’s primary purpose (quote 14f) while others felt they had capitalized on collective brainstorming to improve the interventions approach (quote 14a).

#### Failure to scale-up

When asked to talk about experiences deploying pilot studies that had not been successfully scaled, investigators focused on a combination of multiple factors rather than singular events or influence, citing lack of sufficient effects, low participant reception, shifting funding priorities, limited researcher capacity and lack of large-scale funding for a given topic (quote 14a–14c).

## Discussion/conclusion

To better understand the process of scaling-up early-stage trials, we conducted interviews with 24 investigators who had a published pilot study and subsequent trial on topics related to childhood obesity. Investigators reported that pilot studies were used to preemptively address challenges such as engaging key stakeholders, establishing trial parameters and measurement logistics, nonetheless, these same elements were identified as reasons scaled-up trials were less successful. These findings suggest that, while pilot studies may result in favorable trial-related parameters and evidence of preliminary efficacy, this evidence may not accurately reflect the conditions encountered during the implementation and evaluation of the subsequent scaled-up intervention.

The current study builds on previous findings indicating some components of early-stage studies have a high potential to produce exaggerated or inflated effects which diminish during subsequent, scaled-up iterations [12, 14]. Components which were previously identified through quantitative meta-epidemiological methodology [12], such as delivery agent bias and target audience bias, were confirmed qualitatively by our participants. Major causes of inflated effects suggested by investigators included high levels of oversight in pilot studies (e.g., researcher support) and utilizing biased or highly motivated samples. Given the evident alignment between investigator-reported elements and patterns identified in the meta-epidemiologic study, the data suggests that investigators can choose to minimize or eliminate inflated effects when executing pilot studies. In doing so, the results of pilot studies could provide better evidence upon which decisions about scaling or re-piloting can be made.

Findings from this study suggest that potential conflict may be present between the need to obtain funding and the need to assess key uncertainties when piloting interventions. Investigators expressed that obtaining funding for a larger intervention was a key definition of success. To attain funding, investigators may introduce artifacts within their pilot studies that lead to inflated effects and it is these inflated effects that may be viewed more favorably from a funding perspective. Minimizing potentially inflated effects obtained from pilot studies was not mentioned as a key consideration when executing early-stage work by the investigators even though they recognized inflated effects often occur. Early-stage studies that minimize artifacts associated with inflated effects, such as ensuring the sample in the pilot study matches the sample in the larger-scale trial, have a smaller reduction in their effectiveness when scaled and a greater likelihood of demonstrating statistically significant effects in the larger-scale trial [12, 14]. With funding as a key marker of success, investigators may knowingly or unknowingly design and execute their pilot study to ensure this success, rather than designing/executing the pilot study to understand whether or not the intervention can be adequately scaled while retaining some of its initial effectiveness.

The conflict between piloting for key uncertainties and the need to obtain funding may also be exasperated by the common two-step process for intervention development—initial piloting with small sample sizes followed by a larger-scale trial with a substantially larger number of participants. This may inadvertently force investigators to choose between fostering evidence of effects over addressing key uncertainties related to feasibility and acceptability. In other words, investigators may only have a single opportunity to establish the “value” of the intervention being piloted. Funding structures may add to this conflict by offering few mechanisms that support a multi-phased developmental approach for early-stage studies which offer the opportunity to pilot and refine an intervention in progressively larger sample sizes, as opposed to conducting a single small study and using this as the only basis for which to judge the readiness of an intervention to be tested on a much larger-scale [14, 30]. Thus, the interventionists in this study may be expressing their opinions of success as a reflection of how current funding structures are established rather than whether the process of an initial pilot followed by a larger-scale trial is the most optimal way to establish evidence for scaling.

Existing models for scaling behavioral research describe an iterative sequencing of formative work which may help address this concern, though they vary in the number and purpose of each specified step or phase of the process [18]. Similarly, our interviewees describe using a diverse sequence and size of formative studies to inform later larger-scale trials. Some investigators considered qualitative studies a “first step” while others began with smaller interventions or measurement studies. Some would not initiate pilot studies without initial confidence in the efficacy of the intervention. Others expressed a preference for “tinkering” along the way, using multiple smaller, unpublished intervention studies prior to more formal pilot work. This lack of consensus on sequencing formative work may contribute to some of the variability in pilot study utility. Clarifying the role of formative qualitative work and smaller scale “pre-pilot” studies in the development of later, more formal pilot studies may assist researchers in standardizing and streamlining procedures and protocols, and therein, codifying the delivered intervention and stabilizing observed effects [22]. This process can be linear, though it can also be recursive with investigators piloting interventions several times (i.e., re-piloting) when initial effects were insufficient or recruitment and retention were poor.

Investigators who perceived the results of their pilot studies to be lacking, often re-piloted or abandoned the next stage of their intervention development. However, those who felt their pilot results were promising often adapted elements of their interventions in their larger trial, sometimes involving a major reworking of the intervention. These reworkings included changing the intervention protocol and/or utilizing different locations, settings, or delivery agents. Investigators often perceived the process of interpreting pilot-produced evidence as nebulous, involving both quantitative markers of preliminary efficacy and process measures such as acceptability collected via mixed-methodology. This perspective of interpreting feasibility markers alongside preliminary signals of efficacy is reflective of changes in the field of behavioral research where investigators suggest testing for statistical effects in pilot studies is no longer recommended [31, 32].

There a several imitations in the current study. First, we purposively sampled only individuals who had conducted and published pilot research of behavioral interventions and subsequent scaled-up trials of the same or similar intervention. While early-stage research is widely used to develop behavioral interventions, there are a number of larger-scale behavioral trials that do not explicitly reference early-stage research that informed the intervention. Additionally, there are a large number of published pilots that do not inform later scaled-up trials. The viewpoints of authors who did not explicitly cite pilot trials and authors who have not scaled-up pilot trials are therefore not represented. Second, response bias may be present among the interviewees. Investigators who felt more comfortable discussing their early-stage work, those who had more positive experiences executing pilot studies, or who spent more time engaging in reflection on preliminary work, may have been more likely to participate. Though the 24 participants interviewed represent 63% of the entire eligible population of PIs, the potentially unique perspectives of non-respondent investigators are not represented. Additionally, it is possible that the inclusion of authors as participants may have skewed findings (AO, DL, RJ). However, it should be noted that the conceptual/theoretical approach employed for this study allowed for the inclusion of participant-authors (AO, DL, RJ) and intentional, pre-planned methodological steps were employed to ensure the trustworthiness of the data. Lastly, it was not practical to conduct interviews face-to-face as the participants were located across four continents. Thus, contextual information like body language were not collected.

In conclusion, our study suggests that careful planning during the early-stage testing of an intervention can lead to pilot studies that provide important information about whether an intervention should be scaled-up. This could be achieved by supporting behavioral interventionists in designing pilot studies that closely mimic the condition encountered at scale. Additionally, guided interpretation of pilot-produced information could assist interventionists in making prudent, informed decisions regarding the necessity and appropriateness of re-piloting their interventions prior to scaling such as transparency in the rationale and a priori progression criteria in pilot studies. High-quality pilot studies have the potential to inform effective, impactful interventions to address important public health problems. However, the design, execution and interpretation of high-quality pilot studies should not be assumed to be self-evident or intuitive but rather the result of careful planning, execution, and thoughtful interpretation.

## Supplementary Information

**Additional file 1.** Semi-Structured Interview Guide.

**Additional file 2.** A Priori Themes and Emergent Themes.

## Data Availability

Data will be made available upon reasonable request to the corresponding author.
