# Correction to: Early-stage studies to larger-scale trials: investigators’ perspectives on scaling-up childhood obesity interventions

**DOI:** 10.1186/s40814-022-01047-7

**Published:** 2022-04-23

**Authors:** L. von Klinggraef, R. Dugger, A. D. Okely, D. Lubans, R. Jago, S. Burkart, R. G. Weaver, B. Armstrong, C. D. Pfedderer, M. W. Beets

**Affiliations:** 1 Arnold School of Public Health, University of South Carolina, 921 Assembly Street, Room 130, Columbia, SC 29205 USA; 2 School of Health and Society, University of Wollongong, Wollongong, Australia; 3 Priority Research Centre for Physical Activity and Nutrition, University of Newcastle, Callaghan, Australia; 4 Centre for Exercise, Nutrition & Health Sciences, School for Policy Studies, University of Bristol, Bristol, UK

**Correction to: Pilot Feasibility Stud 8, 31 (2022)**

**https://doi.org/10.1186/s40814-022-00991-8**

Following publication of the original article [1], the authors identified an error in Table 1. The correct table is given below.

**Table 1:** Thematic analysis results

Theme	Definition	Exemplary Quote	
**I. Conceptualization of Intervention**			
Gathering Information	Conduct an initial exploration of potential interventions including literature reviews and qualitative inquiry	1a	“I like to make sure that I'm informed by investors in the program. So if it’s a school-based program, I’d be speaking to the school, the children, the parents, or people who are experts in that area, to make sure that the program is what they are looking for, what they need.” ID6
		1b	“Looking at the evidence based behavioral strategies techniques that have been shown to be useful within that specific population, you know, highlighting the sort of the mechanisms of actions.” ID23
Measurement Logistics	Will study measures result in a sufficient amount of quality data.	2a	“Do we want to do DEXA scans in this particular trial? Will people accept these DEXA? Let's try it. Let's see. Will they wear FitBits? Will they wear them 24 hours? Will they wear them at night so we can monitor what's going on at sleep? You're getting pilot usability and feasibility feedback.” ID19
		2b	“You use pilot studies to check your measurements, to be sure you can do them. The measures I do are more complicated to deliver because we’re not located near the participants.” ID5
Trial Parameters	Will intervention and study design be well-received by the target population and whether study can be executed with sufficient fidelity to produce a valid test of intervention efficacy, feasibility, and acceptability	3a	“Can you recruit at the rate that you think? Will people deliver the intervention? Can we assign people to control? And is that acceptable? Testing all of those different parameters.” ID19
		3b	“You're tracking a whole range of methodological considerations, testing the actual program or intervention, and it gives you an opportunity to see the acceptability, the feasibility, of delivery, the quality” ID17
Mirror Larger Trial	Intentional consideration of the alignment between preliminary studies and future, larger trials of the same/similar intervention	4a	“The pilot study has to be conducted exactly the way the future large-scale study would be conducted. Naturally, it has to be conducted on a smaller scale because of the funds available at that time.” ID22
		4b	“In terms of mimicking what would actually happen in a definitive trial … sometimes you can’t know what will happen until you’ve made these mistakes.” ID10
**II. Interpretation of Pilot Study Results**			
Process Implementation Measures	Measures of process implementation including fidelity and dose, as well as indicators of participant engagement such as recruitment, retainment, and qualitative feedback.	5a	“We knew we could recruit and retain. We knew the kids liked it, the staff were reporting, the engagement was high. That was key because the intervention required quite high engagement levels. We could get good physical activity data with high compliance. That was a big tip.” ID13
		5b	“I want to see some data that says, number one, people come and number two, people will stay in till the end.” ID9
Indicators of preliminary efficacy	Markers used to assess potential efficacy such as trends in primary outcomes, effects of theorized mediators, and tests of statistical significance.	6a	“Pilot studies can provide some evidence to move forward, but they are often underpowered to detect differences. So, I think pilot trials have a place, but I don’t think it should be the deciding factor.” ID2
		6b	“What we did have was some pre and post data, and a hunch that we liked it” ID4
		6c	“A pilot study has to provide some evidence of efficacy. The suggestion to conduct a feasibility or pilot study and not worry about the outcome … we could waste a lot of resources if we had that view.” ID14
		6d	It’s a bit of a conundrum. I do think that you can falsely say ‘oh, well we didn’t show an effect in our preliminary efficacy because the sample size was small, and the timing was off’. ID24
Inflated effects	Aspects of pilot studies that may inflate indicators of efficacy.	7a	“The participants in a pilot study are the cream of the crop, and when you’re doing the larger study those people have already participated.” ID5
		7b	“In the pilot study we had teachers delivering intervention and in the main trial we went to the core non-degree staff delivering the intervention. So that probably inflated the effect of the pilot.” ID21
		7c	“There’s no point doing it, showing all this promise from a pilot study when many of the components have no chance of going to the next stage.” ID17
**Concept III: Scaling Piloted Interventions**			
Re-piloting Interventions	Engaging in more than one round of preliminary testing to enhance intervention effects or address identifed obstacles	8a	“We had some effects, but we thought we could make them better. So we added more teaching practice and we revised the activities to make sure that they maximized physical activity opportunity.” ID3
		8b	“The participants just weren't engaging beyond our initial clinic visit and that suggested, to me, that what we had developed was probably not going to be sufficiently engaging.” ID15
		8c	“If the core aim of what you are aiming to achieve has to change substantially, then I think you should reconsider whether it warrants another pilot.” ID15
Strategies	Strategies to ensure a larger trial was completed	9a	“We had very good partnerships. We were embedded in the local school boards and they saw it as very useful from both a health and an educational point of view and the larger scale trial was really just a larger scale longer version of the pilot study.” ID20
		9b	“We were mindful that the teachers in the intervention schools may not be as motivated as those in the pilot schools. So we had to give them some flexibility around how they would be implementing the intervention.” ID18
		9c	“As we moved from a pilot study to a larger-scale trial, it was not possible to get one person to train all the teachers, so we kind of had to move to a train the trainer model.” ID10
Funding	Choices made to conduct, and publish, preliminary work were based upon investigators perceptions of the difficulty, and value of the preliminary work as it related to obtaining additional funding.	10a	“The primary outcome for our larger study might be the secondary outcome for the pilot study. In our pilot stud, we might look at a behavioral outcome rather than a clinical outcome because we know that we know we are not going to show anything on the clinical outcomes” ID15
		10b	“The big incentive of having good pilot data is getting funding.” ID7
		10c	“I don’t do a pilot study unless I know it’s worthwhile.” ID6
		10d	“Journals don’t want to publish no results, and pilots are often null.” ID2
		10e	“If you focus on publications quality, then pilot study publications might not be the highest quality publications. They might not be getting the best or highest ranked journals” ID3
Adaptions	Identification of obstacles with study design or intervention protocol and brainstorms ways to ameliorate the identified problems	11a	“We received feedback from the first feasibility trial and they said this certain activity is really good. So we focused on the ones that worked really well and then the feedback that we got from the second study was that these didn’t work so well.” ID3
		11b	“The other key difference is that based on our pilot study intervention was having more of an effect on children with a propensity for overweight or obesity. So in the larger scale study, we focused specifically on those children.” ID7
		11c	“If people didn't adhere to what you're doing, if you couldn't recruit enough people, if satisfaction was a complete fail … .I would have probably done adjustments along the way to make the pilot successful, but if I hadn’t, then okay, I’d need to pilot this again.” ID24
		11d	“I’d consider whether you have differential dropout or follow-up between the intervention and control groups. And if you do, then you will need to see how best to address that before moving on to the full trial.” ID13
Challenges	Challenges encountered when scaling pilot studies	12a	“In the pilot we had a small amount of group of teachers who were very motivated and who want to bring about a change but in our larger trial we had multiple teachers, multiple schools. So staff felt like their principal or their head of department had a sort of top-down approach.” ID18
		12b	“The control group was more powerful than we expected and the intervention didn’t look that meaningful next to the control group. That made it challenging argue for the larger study.” ID5
		12c	“For the larger study, going into low income schools in more disadvantaged areas, recruiting, retaining, and gauging is just so much harder.” ID14
**Concept IV: Reflection**			
Lessons learned	The process of executing a pilot study, and subsequently larger trial, provided investigators with valuable insight	13a	“We’ll sit there in a room for a day, go through things, and ask ‘What do you think?’ and then say ‘oh well that didn’t work, this didn’t work.’ So we put our minds together and, use those lessons from our other trials, to move forward.” ID6
		13b	“I think you’ve got to have the methodological questions built in and you might also want to do a much more detailed evaluation of behavior changes rather than just focusing on the outcome which you know in a larger trial you might not be able to collect.” ID4
		13c	“My lesson was to not sit there too long and try to do multiple feasibility studies.” ID11
		13d	“I learned a lot about just this notion of the need to not overreach and not overthink what it is that you can do.” ID19
		13e	“We know now that a big study has to be very simple.” ID12
		13f	“My colleagues were proposing that we shouldn’t even test the behavioral intervention. They wanted to test use a sham intervention to test other components, such as recruitment, and blinding, and different factors. But I argued that I wanted to match the methods of the feasibility trial” ID5
Failure to scale	Combination of factors that blocked a larger scale trial based upon pilot results	14a	“The pilot studies did not go on to a full trial because the effects were not substantial enough, and not consistent enough.” ID3
		14b	“The larger trial never materialized because we were unable to get funding. We just felt like the evidence wasn’t as convincing as we would have liked.” ID18
		14c	“I wouldn't take it forward to full trial because it just wasn't well received. We didn't get good engagement with clinicians and we struggled to recruit, despite spending a lot of time and a lot of resource.” ID15

The original article [1] has been updated.
