# Supplementary material for: Early-stage studies to larger-scale trials: investigators’ perspectives on scaling-up childhood obesity interventions
Source: Pilot Feasibility Stud. 2022 Feb 7;8:31. doi: 10.1186/s40814-022-00991-8 (PMC8819854; doi:10.1186/s40814-022-00991-8)
Supplement: Supplementary file 1 — Additional file 1. Semi-Structured Interview Guide. [file 40814_2022_991_MOESM1_ESM.docx]

Additional File 1: Semi-Structured Interview Guide

The following semi-structured schedule of questions was used. Additional questions were posed as prompted by participants answers.

1. **Question:** Across your career, how many pilot studies have you been involved with and how many of these have progressed to a larger trial?
2. **Question:** What do you think should be the purpose of a pilot study of a behavioral intervention?
3. **Question:** What do think are the features/characteristics of a high-quality pilot study that achieves that purpose?

In thinking about your pilot study and larger-scale trial (refer to referenced studies)…

1. **Question:** What were the challenges you experienced in taking your pilot study to a larger-scale trial?
2. **Question:** What evidence from your pilot study did you use to make the decision to progress to a larger trial?

**Question:** Please describe the strategies that helped you to take your pilot study to a larger-scale trial?

1. **Question:** What lessons did you learn (either addressable or not addressable) from your pilot and how did you apply these lessons to the larger-scale trial? Were there any lessons you were unable to address in your pilot?
2. **Question:** What changes, if any, did you make to the larger-scale trial based upon the results of the pilot study? These can include changes to the intervention, research design, measures, etc…
3. **Question:** Have you conducted other pilots that did not go to a full-scale trial? If “yes”, why did the pilot not go to a full-scale trial?
4. **Question:** In thinking about the scientific field as a whole, what types of incentives do you believe exist for producing and publishing pilot studies?
