# Supplementary material for: Early-stage studies to larger-scale trials: investigators’ perspectives on scaling-up childhood obesity interventions
Source: Pilot Feasibility Stud. 2022 Feb 7;8:31. doi: 10.1186/s40814-022-00991-8 (PMC8819854; doi:10.1186/s40814-022-00991-8)
Supplement: Supplementary file 2 — Additional file 2. A Priori Themes and Emergent Themes. [file 40814_2022_991_MOESM2_ESM.docx]

Additional File 2: A Priori Themes and Emergent Themes

A Priori Themes:

- Purpose of a pilot
- Feature/characteristics of a pilot to meet that purpose
- Use of statistics in deciding to move forward
- Similarities between pilot and larger scale trial intervention
- Considerations for moving forward – decision rules
- When to re-pilot an intervention
- Challenges experienced in taking their pilot to a larger scale trial
- Incentives to produce and publish pilot studies
- Elements introduced in pilots that cannot be introduced in larger scale trials that produce exaggerated effects – what are they and solutions?

Emergent Themes

- Role of Funding
- Role of Publication
- Role of Students
